# Structural Characterization, Cytotoxicity and Microbiological Activity of One-Step-Synthesized RGO/AuNPs Nanocomposites

**DOI:** 10.3390/ma18194464

**Published:** 2025-09-25

**Authors:** Boris Martinov, Dimitar Dimitrov, Tsvetelina Foteva, Aneliya Kostadinova, Anna Staneva

**Affiliations:** 1 Department of Biotechnology, University of Chemical Technology and Metallurgy, No. 8, Kliment Ohridski Bvd, 1797 Sofia, Bulgaria; tsvetelina.foteva@uctm.edu; 2 Department of Silicate Technology, University of Chemical Technology and Metallurgy, No. 8, Kliment Ohridski Bvd, 1797 Sofia, Bulgaria; dimithar@uctm.edu (D.D.); ani_sta@uctm.edu (A.S.); 3 Lipid-Protein Interactions, Institute of Biophysics and Biomedical Engineering, Bulgarian Academy of Sciences, Acad. G. Bonchev Str. Bl. 21, 1113 Sofia, Bulgaria; aneliakk@yahoo.com

**Keywords:** gold nanoparticles, antimicrobial nanoparticles, cytotoxicity, reduced graphene oxide, nanocomposite, graphene

## Abstract

This study presents a green, single-step method for synthesizing nanocomposites based on reduced graphene oxide (RGO) and gold nanoparticles (AuNPs), using sodium citrate as a mild reducing and stabilizing agent. AuNPs were generated from chloroauric acid (HAuCl_4_) directly on the surface of graphene oxide (GO), which was simultaneously reduced to RGO. Structural characterization via Transmission Electron Microscopy (TEM), High Resolution TEM (HRTEM) and Selected Area Electron Diffraction (SAED) confirms spherical AuNPs (10–60 nm) distributed on RGO sheets, with indications of nanoparticle aggregation. Dynamic Light Scattering (DLS) and zeta potential analysis support these findings, suggesting colloidal instability with higher RGO content. Biological evaluation demonstrates dose-dependent cytotoxicity in HaCaT keratinocytes, with IC_50_ values (half maximal inhibitory concentration) decreasing as RGO content is increased. At moderate dilutions (1–25 µL/100 µL), the composites show acceptable cell viability (>70%). Antibacterial assays reveal strong synergistic effects against *Escherichia coli*, *Staphylococcus aureus*, and *Bacillus subtilis*, with sample RGO/Au 0.500/0.175 g/L showing complete *E. coli* inhibition at low Au content (0.175 g/L). The composite retained activity even in protein-rich media, suggesting potential for antimicrobial applications. These findings highlight the potential of RGO/AuNPs composites as multifunctional materials for biomedical uses, particularly in antimicrobial coatings and targeted therapeutic strategies.

## 1. Introduction

Graphene-based materials, particularly graphene oxide (GO) and reduced graphene oxide (RGO), have garnered significant attention in recent years due to their exceptional physical, chemical, and biological properties [1]. GO is well known for its large surface area, electrical conductivity, and biocompatibility, enabling its use in biomedical applications such as drug delivery, antimicrobial coatings, and tissue engineering [2]. Gold nanoparticles (AuNPs), valued for their chemical stability, plasmonic properties, and ease of surface modification, are widely used in drug delivery, diagnostics, and environmental sensing [3,4]. Combining graphene with AuNPs results in multifunctional nanocomposites that integrate the antibacterial and electrochemical properties of both materials. These hybrids exhibit enhanced Reactive Oxygen Species (ROS) generation and electrochemical activity, leading to improved antibacterial efficacy [5,6]. Turcheniuk et al. demonstrated that AuNPs combined with RGO exhibit greater antibacterial activity than either component alone [7]. In contrast, other studies show that high Au content (>200 µg/mL) is often required to achieve partial antibacterial effects in similar composites [8]. A common synthesis route for AuNPs is the citrate reduction method introduced by Turkevich et al., where sodium citrate functions as both reducing and stabilizing agent [9]. The formation of AuNPs generally proceeds through a two-step process:

Au^3+^ + reducing agent → Au^0^ → nAu^0^

As a starting material, tetrachloroauric acid (HAuCl_4_.nH_2_O) has been used. The reducing agents can vary: hydrogen and hydrogen-containing compounds (e.g., tetrahydroborates), phosphorus, tin chloride, sodium citrate, hydrazine, alcohols, ethylene glycol, starch, glucose, ascorbic acid, and others. The reduction is carried out in the presence of stabilizing organic substances—ligands (L), which can also impart catalytic properties, bio-recognition, transport, and other characteristics to the nanoparticles. The second stage corresponds to the formation and growth of nanoparticles, during which the ligands do not formally participate in the process, but their presence affects the size and shape of the particles:

nAu^0^ → [Au^0^]_n_ + mL → [Au^0^]_n_L_m_

These steps correspond to the initial reduction of gold ions, followed by nanoparticle nucleation and growth, which are influenced by the ligand environment [10].

Graphene-AuNP nanocomposites are promising in therapeutic technologies such as cancer photothermal therapy, due to their optoelectronic tunability and potential for functionalization [11,12,13]. Their combined mechanical and oxidative effects enhance antibacterial performance by disrupting membranes and promoting ROS-related damage [7,14].

Surface functionalization has also been shown to enhance their bactericidal properties and support targeted cancer therapy applications [15,16]. These mechanisms align with previous studies on graphene-metal systems [13,17]. Furthermore, the improved electron mobility of graphene oxide also contributes to the effective distribution of ROS, enhancing the overall bactericidal effect [18]. The reduction of both GO and Au^3+^ with a single green reagent, such as sodium citrate, enables a simplified one-pot synthesis. Wan et al. demonstrated the effectiveness of citrate in GO reduction [19]. Thus, the reduction of both Au^3+^ and GO can be achieved using the same reducing agent.

In this study, we present a green, one-step method for synthesizing RGO/AuNP composites via simultaneous reduction of Au^3+^ ions and GO using sodium citrate. The resulting materials exhibit well-dispersed gold nanoparticles, enhanced antibacterial activity, and maintained biocompatibility. Compared to previous studies, our approach achieves these effects at lower gold content and through a simpler synthesis, highlighting the composites’ potential for biomedical applications such as antimicrobial coatings and targeted therapies.

## 2. Materials and Methods

### 2.1. Synthesis of Au Nanoparticles

**Figure 1:**
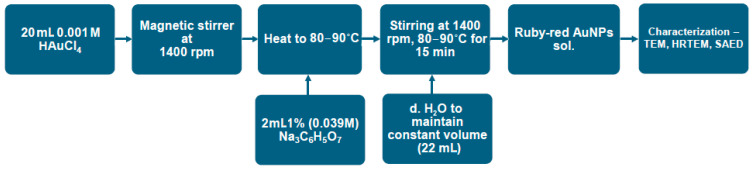
Synthesis route for AuNPs until a ruby red solution (sol.) is obtained.

The synthesis of gold nanoparticles using the citrate method was performed according to the scheme presented in Figure 1. A 20 mL 0.001 M solution of chloroauric acid (HAuCl_4_) was prepared in a 100 mL heat-resistant beaker. The solution was heated to 80–90 °C with continuous stirring with a magnetic stirrer (1400 rpm). Once the target temperature had been achieved, 2 mL of a 1% (0.039 M) solution of sodium citrate (Na_3_C_6_H_5_O_7_) was added. Heating and stirring continued until the solution turned a ruby-red color (approximately 15 min). During the reaction, the volume of the solution was kept constant at 22 mL by adding small portions of distilled water using a 5 mL pipette. The color change in the solution indicated the occurrence of chemical and structural transformations in the system. Once the solution had reached a final ruby-red color, characterization of the obtained Au nanoparticles was immediately initiated.

### 2.2. Synthesis of RGO/AuNPs Composites

**Figure 2:**
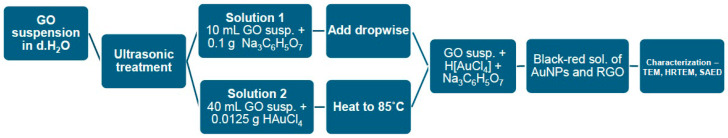
Synthesis route for obtaining RGO/AuNPs composite using GO suspension (susp.).

Figure 2 presents the route of synthesis for the composite material. In a heat-resistant beaker, 50 mg of GO and 100 mL of distilled water were added. Ultrasonic treatment was performed for 30 min (80% power). The suspension was then divided:

- Solution 1. To 10 mL of the GO suspension, add 0.1 g of Na_3_C_6_H_5_O_7_.
- Solution 2. To 40 mL of the GO suspension, add 0.0125 g of HAuCl_4_. Heat to 85 °C on a magnetic stirrer.

Upon reaching 85 °C, 4 mL of Solution 1 was added dropwise to Solution 2 with constant stirring (1400 rpm). After maintaining these conditions for 10 min, the solution is slowly cooled to room temperature to complete the reduction processes and obtain a black-red aqueous suspension of the RGO/AuNPs composite.

Using an analogous approach, composites with varying ratios of RGO to AuNPs were obtained (Table 1).

### 2.3. Characterization Techniques

#### 2.3.1. TEM Analysis

The TEM analysis of the synthesized AuNPs/RGO composites was performed using an HR STEM JEOL JEM 2100 apparatus at the Bulgarian Academy of Sciences (BAS). This analysis is used to observe structural morphology of the gold nanoparticles and RGO sheets, as well as their size. In addition, Selected Area Electron Diffraction (SAED) was performed to determine crystal lattice parameter and interplanar spacing of the obtained AuNPs.

#### 2.3.2. Dynamic Light Scattering

Dynamic light scattering (DLS) was used to determine the average size, polydispersity index (PDI), and apparent zeta potential values of the composites. These parameters are connected to the cytotoxicity against eukaryotic cells as well as antibacterial activity against Gram-positive and Gram-negative bacteria. The measurements were conducted using a Zetasizer Nano ZS analyzer (Malvern Instruments, Malvern, UK) equipped with a U-type cell containing gold electrodes. The DLS profiles of RGO composites were obtained at a concentration of 10 µL, at 25 °C after preparing the dispersions. Each sample was measured three times per experiment.

### 2.4. Cytotoxicity and Biocompatibility

In order to study cell survival, the HaCaT keratinocyte cell line (Bulgarian national collection for microorganisms and cell cultures) was chosen as a model since the skin is the first to be exposed to external influences. The experiment was used to assess cytotoxicity and to determine the IC_50_ of the investigated nanomaterials. The eukaryotic cells were maintained under standard conditions in a humidified atmosphere with 5% CO_2_ at 37 °C in Dulbecco’s Modified Eagle’s Medium (DMEM) (Sigma) medium, supplemented with 10% fetal bovine serum (FBS) (BioWhittaker™) and 1% (*v*/*v*) antibiotic-antimycotic solution (penicillin 100 U/mL, streptomycin 100 µg/mL, and amphotericin B 0.25 µg/mL; BioWhittaker™).

For cytotoxicity assessment, crystal violet (Sigma-Aldrich) staining and microscopic observation were performed. RGO composites at varying dilutions were added to 24-well plates. Subsequently, HaCaT cells were seeded at a concentration of 1 × 10^5^ cells/mL and treated with different RGO/Au nanomaterial dilutions ranging from 1 to 80 µL in a total volume of 100 µL. After 24 h, cytotoxicity was evaluated via the crystal violet assay, and cell morphology was observed by bright-field microscopy. The residual cell monolayer was washed with phosphate-buffered saline (PBS) and fixed with 4% paraformaldehyde in PBS for 15 min. After that, plates were washed with water, and 200 µL 0.1% crystal-violet solutions were added to every well. After 20 min incubation at room temperature, the plates were washed with water, and the protein-bound dye (corresponding to the cell number) was extracted with 200 µL 10% acetic acid. The optical density values were read on a microplate reader (EPOCH UV/VIS Spectrometer) at 570 nm, and the number of vital cells was calculated as a percentage of their total amount. Invert microscope pictures were taken using supplied with a digital camera DV-130, XDS-2A microscope, China. Six biological replicates were performed for each composition.

### 2.5. Antimicrobial Activity

Colonies of *E. coli* ATCC25922 and *S. aureus* ATCC 25923 were preliminarily incubated overnight. A total of 100 μL of the obtained growth was introduced in 5 mL Meat Peptone Broth (MPB). After 5 h the suspension was diluted to 0.5 McFarland standard with saline. The Au/RGO samples were sonicated and diluted to 2×, 4×, and 8× with distilled water.

#### 2.5.1. Broth Dilution Method (Saline-D. Water)

A 96-well plate was filled with 100 μL 0.5 McF of the two bacterial cultures. A total of 100 μL of the initial AuNPs/RGO suspension was added to three wells each of *E. coli* and *S. aureus*, thus obtaining 2× dilution. 100 μL of the previously prepared 2× dilution was added to the next 3 wells, thus obtaining 4× and so forth up to 16× for samples 1, 2, and 3. (Table 1). For samples 4 and 5, dilutions up to 8× were used.

The same procedure was repeated, but this time after reaching the exponential phase of growth, the biomass was centrifuged at 10,000 rpm and extracted from the growth medium, then flushed with saline three times. Finally, a 0.5 McF suspension was prepared and treated with the nanocomposite suspensions in an analogous way.

At hours 2, 20, and 30, 5 μL from the wells was added to a segmented dish and incubated for 24 h to assess the level of inhibition.

#### 2.5.2. Agar Diffusion and Modified Spot-Test Methods

Melted and cooled MPB agar was inoculated with 0.5 McF exponential phase cultures and poured into Petri dishes. Wells were carved into half of them, and 50 μL of the prepared composite dilutions were added. After cooling for 2 h, the dishes were incubated at 37 °C for 24 h. For the modified spot-test method, 5 μL of the nanocomposite samples were added to the cooled agar directly and incubated at 37 °C for 24 h.

Further studies with *B. subtilis* culture were conducted with 0.5 McF. The following procedure was used:

- Surface inoculation (100 µL in Petri dishes on Nutrient Broth agar (NB-agar));
- Incubation at 30 °C for 30 min;
- Loading of filter discs with 20 μL of synthesized solutions.
- Drying at 30 °C for 30 min;
- Incubation of the Petri dishes at 30 °C for 24 h.
- All antibacterial experiments were performed in triplicate.

### 2.6. Statistical Analysis Method

Data was analyzed using one-way analysis of variance (ANOVA) to determine statistically significant differences between groups. The analysis was performed using GraphPad. A *p*-value of less than 0.05 (*p* < 0.05) was considered statistically significant. Results are expressed as mean ± standard deviation (SD), based on at least three independent experiments (for the cytotoxicity study, *n* = 6).

## 3. Results

### 3.1. TEM Analysis Results

#### 3.1.1. Gold Nanoparticles

**Figure 3:**
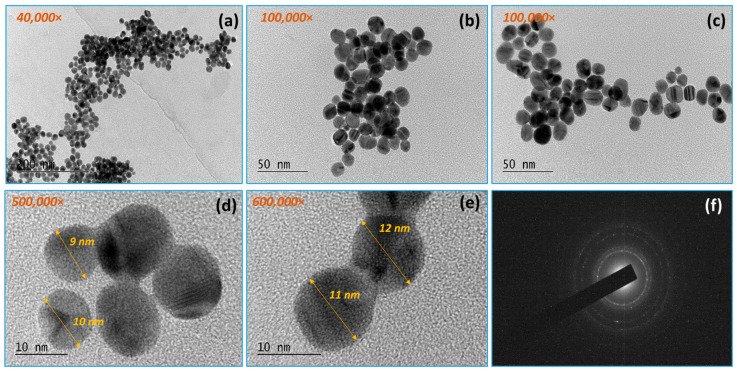
TEM analysis of gold nanoparticles in different areas of sample magnifications: (**a**) 400,000×; (**b**,**c**) 100,000×; (**d**) 500,000×; (**e**) 600,000×; (**f**) SAED (selected area electron diffraction), where the bright concentric rings confirm the polycrystalline structure of the Au nanoparticles, and the central dark shadow is caused by the beam stopper.

In the case of the separately synthesized AuNPs, TEM analysis (Figure 3) confirms well-shaped spherical Au nanoparticles with a size of about 9–12 nm. From observation under different magnifications, it is evident that the gold nanoparticles are approximately uniform in size, round in shape, and well distributed in volume. No aggregation of the gold nanoparticles was observed.

#### 3.1.2. RGO/AuNPs

**Figure 4:**
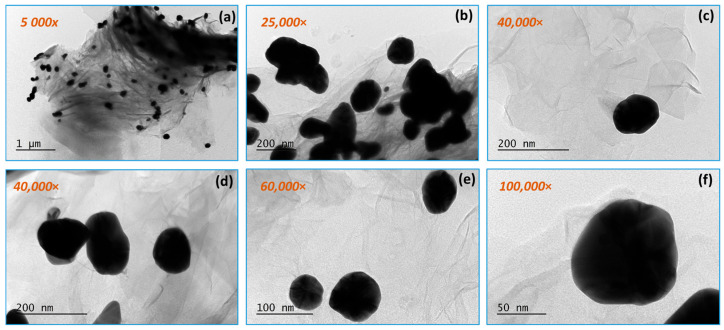
TEM analysis of RGO/Au 0.500/0.175 g/L nanocomposite at magnifications (**a**) 5000×; (**b**) 25,000×; (**c**,**d**) 40,000×; (**e**) 60,000×; (**f**) 100,000× in selected areas.

**Figure 5:**
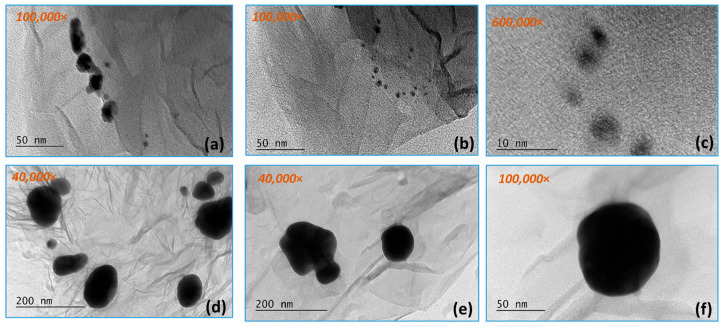
TEM analysis of RGO/Au 0.500/0.175 g/L nanocomposite at magnifications (**a**,**b**) 100,000; (**c**) 600,000×; (**d**,**e**) 40,000×; (**f**) 100,000× in selected areas.

Figure 4 and Figure 5 present TEM micrographs of the obtained graphene composite with the participation of gold nanoparticles at different magnifications. The silk-like sheet structure of the graphene layers can be observed. Gold nanoparticles with a size of 50–80 nm are distributed along the RGO surface. The gold nanoparticles vary in size over a fairly wide range (from 8 to 10 nm to 50–60 nm), falling within specifications. This can be explained by the formation of aggregates, which are probably made up of smaller-sized (8–10 nm) gold nanoparticles. There is a tendency for the gold nanoparticles to be located along the edges of the graphene layers, as well as on their surface and between them.

**Figure 6:**
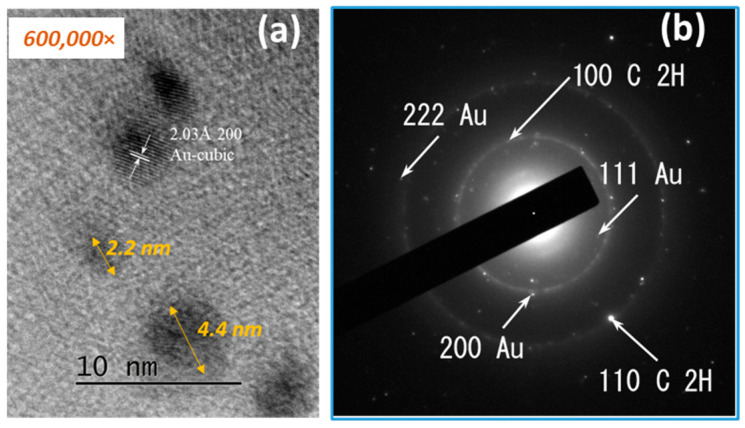
HRTEM image of the RGO/Au 0.500/0.175 g/L composite at 600,000× magnification (**a**), and corresponding SAED pattern (**b**) showing diffraction rings attributed to Au and RGO phases.

Figure 6a shows a high-resolution TEM micrograph showing spherical gold nanoparticles with dimensions below 10 nm. The interplanar spacing measurement of 2.03 Å is in good agreement with the 2.0391 Å 200 of the face-centered cubic phase of gold. The corresponding d-spacing values and Miller indices are summarized in Table 2.

### 3.2. Particle Size Distribution and Surface Charge Results

All five samples from Table 1 have been assessed, and the results are presented in Table 3. The negative Zeta potential signals fast adhesion to eukaryotic cell membranes and weaker interaction with prokaryotes due to their carbohydrate cell walls. This has some predictive power on dampening the expectations regarding antimicrobial activity against Gram-positive bacteria. These results are likely explained by the nature of the citrate ligands. The large apparent size of the particles—ranging between 248 and 589 nm—highlights their tendency to self-adhere and aggregate, further confirmed by the high polydispersity index ranging between 0.3 and 0.850.

### 3.3. Cytotoxicity and Biocompatibility Assay

**Figure 7:**
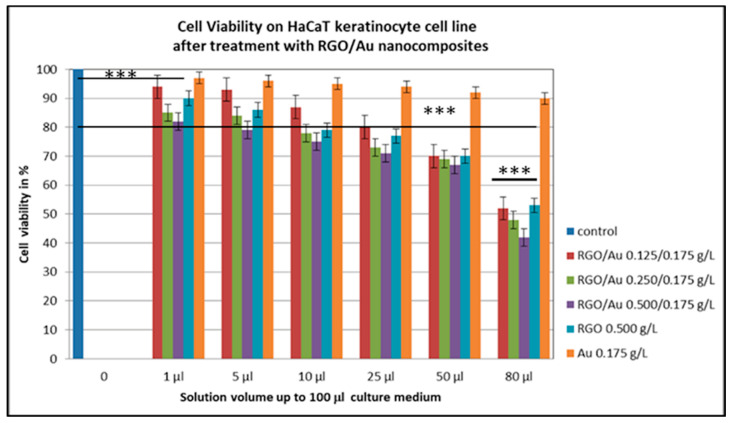
HaCaT keratinocyte cells viability after treatment with different RGO/Au nanocomposites concentrations. Statistical analysis by ANOVA one-way test (*** *p* < 0.001). Data are mean ± SD from 6 biological repeats.

From the cytotoxicity figure carried out with crystal violet, it can be established that at concentrations of 80 µL compared to control, all five RGO/Au nanomaterials were toxic to human skin keratinocytes (*p* < 0.001). Furthermore, at a dilution of 80 µL, all nanomaterials containing RGO/Au in different ratios reduced cell survival by half, i.e., reached the IC_50_ value (Figure 7). The IC_50_ values decreased with increasing RGO concentration, indicating enhanced cytotoxicity. RGO/Au 0.500/0.175 g/L showed the least cell viability of about 43%, RGO/Au 0.250/0.175 g/L also had viability about 52%, while RGO/Au 0.125/0.175 g/L and RGO 0.500 g/L showed about 53–54% (*p* < 0.001). In all dilutions from 1 to 80 µL, AuNPs can be defined as low cytotoxicity, because the cell survival remained high up to 90–95% at 1 µL (*p* < 0.01). From the cytotoxicity test with crystal violet, we can conclude that the most cytotoxic material is RGO/Au 0.500/0.175 g/L. The required dilutions to pass the IC_50_ threshold in the RGO/AuNP samples would be as follows: 77.2 µL for (0.125/0.175 g/L), 72.5 µL for (0.250/0.175 g/L), and 63.0 µL for (0.500/0.175 g/L).

**Figure 8:**
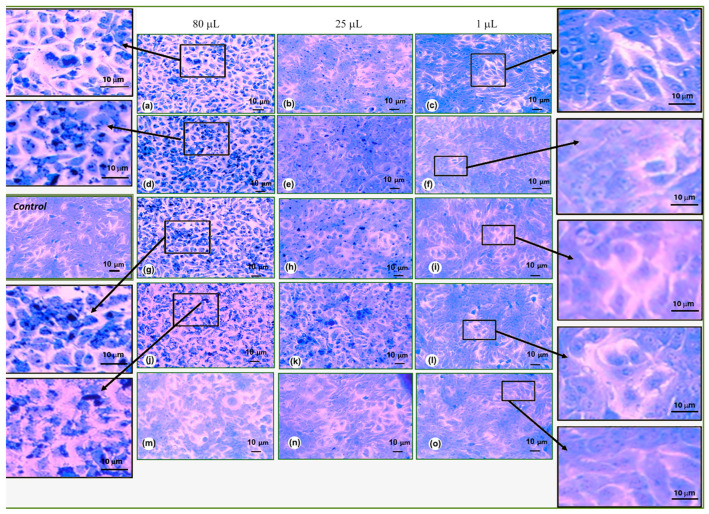
Cell morphology of HaCaT keratinocyte cell line after treatment with different RGO/Au nanomaterial concentrations. RGO/Au 0.125/0.175 g/L (**a**–**c**), RGO/Au 0.250/0.175 g/L (**d**–**f**), RGO/Au 0.500/0.175 g/L (**g**–**i**), RGO 0.500 g/L (**j**–**l**), Аu 0.175 g/L (**m**–**o**). Magnification 200×.

After spectrophotometrically characterizing the cytotoxicity using the crystal violet test, to confirm the results of this quantitative study, we stained the cells with crystal violet and photographed them under a phase contrast microscope. From the microscopic pictures, it was again confirmed that the most cytotoxic is RGO/Au 0.500/0.175 in dilution of 80 µL (Figure 8). The microscopy pictures also clearly show the RGO/Au nanoparticles, which are located near the nucleus. The cells have large intercellular spaces, some are black and highly rounded, which signals cell death. In Figure 8a,d,g, and h, many nanoparticles are also observed in the cytoplasm of the cells, most likely leading to their death. It is noticeable that with a decrease in the dilution of nanomaterials from 25 to 1 µL (Figure 8b,c,e,f,h,i,k,l,n,o), no large clusters of nanoparticles were observed in the cells. Nanoparticles were observed in individual cells at 25 µL up to 100 µL (Figure 8b,h,k,n). Despite the small amounts of nanoparticles in the cells, the latter have a good cell morphology and are relatively densely packed, i.e., the integrity of the cell layer is not disturbed, and the cells communicate well with each other. At a much lower dilution of 5–1 µL, diluted up to 100 µL, nanoparticles are hardly noticeable in the cells. The keratinocytes have a good cell morphology and closely spaced glottis contacts. Dead cells and disturbed cell morphology are not noticeable. This confirms our studies with crystal violet for cytotoxicity and determines the concentrations of the various nanomaterials from dilution 25 µL to 1 µL up to 100 µL as non-cytotoxic.

### 3.4. Antimicrobial Testing

Against *B. subtilis*, some zones of inhibition can be observed. While both samples 2 and 3 show some inhibition, the test highlights sample 2 as superior.

**Figure 9:**
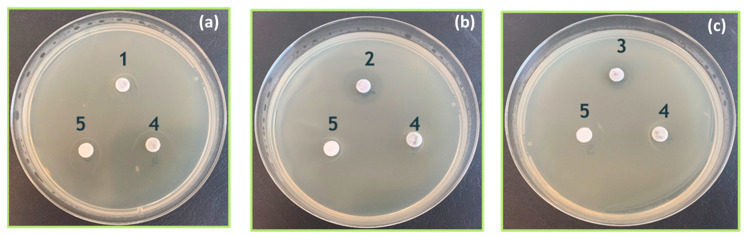
Inhibition zones against *B. subtilis* culture: 0.5 McF of RGO/Au nanocompositeс: (**a**) composition 1 (RGO/Au 0.125/0.175 g/L); (**b**) composition 2 (RGO/Au 0.250/0.175 g/L); (**c**) composition 3 (RGO/Au 0.500/0.175 g/L) when compared to separate components (4—RGO and 5—Au NPs).

The results presented in Figure 9 show that the best antibacterial activity against *B. subtilis* was observed in the following culture: 0.5 McF inoculated with Sample 2—RGO/Au 0.250/0.175 g/L.

While agar diffusion and modified spot test did not reveal inhibition zones against *E. coli* and *S. aureus*, this was not at all the case for the broth dilution method. Samples 3 and 4 showed inhibition up to 16× dilution both in saline and in MPB. Sample 4 shows inhibition up to 2× dilution, while control 5 shows no growth inhibition.

**Figure 10:**
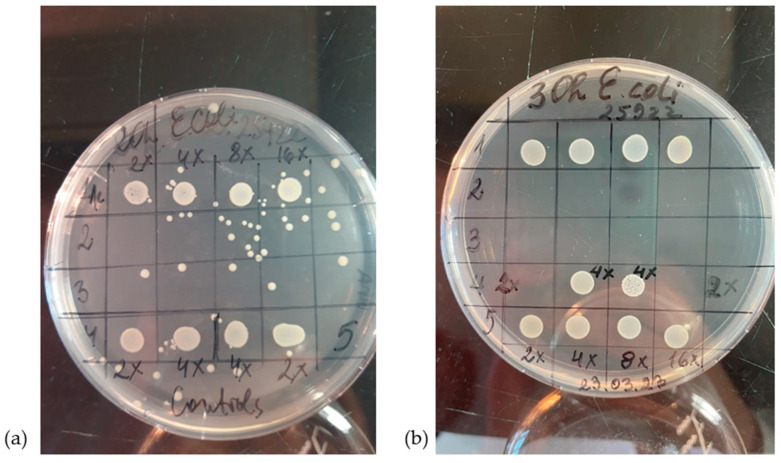
Visual assessment after (**a**) 20 and (**b**)30 hours of incubation.

The experiment was conducted with three technical replicates and two biological replicates for each variant. In the case of bacterial cells washed with physiological saline, mixed 1:1 with distilled water containing freshly sonicated nanoparticles in an ultrasonic bath, inhibition was observed in almost all samples. In samples 1 and 5, there were 100 times fewer cells of both types of bacteria compared to the control—surviving at a level of 10^7^ cells per milliliter (colony-forming units per milliliter, CFU/mL). The strongest effect—a bactericidal effect—was observed with samples 2 and 3, where all tested dilutions showed bactericidal action against *E. coli* American Type Culture Collection 25922 (ATCC 25922). This effect was also clearly visible in the visual assessment of bacterial growth inhibition shown in Figure 10. This can be explained by the thinner cell wall of the Gram-negative bacterium and the lack of a protective effect from the protein medium, as the cells were washed of all remnants of meat-peptone broth in which they had grown (by triple centrifugation and washing with physiological saline). In this case, the Gram-positive bacterium was more resistant due to the thick peptidoglycan layer in its cell wall. For it, a bactericidal effect was observed only with the 2-fold diluted sample 2, while sample 3 reduced the number of microorganisms by 8 orders of magnitude even in a 16-fold dilution.

The results of the conducted study in saline are presented in Table 4, where the number of remaining viable bacteria are shown.

The assay was conducted once more, where dilution was performed with MPB to simulate the performance of the obtained RGO/AuNP composites in living organisms. The experiment was conducted with three technical replicates and two biological replicates for each variant. In the case of the double-diluted nutrient medium (Meat-Peptone Broth: distilled water with nanoparticles, freshly sonicated in an ultrasonic bath), inhibition was observed only with sample 3, and only at nanoparticle concentrations up to 8-fold dilution. The 16-fold diluted sample 3 inhibited *E. coli* ATCC 25922 by a thousand times, meaning three orders of magnitude fewer cells survived the exposure. In the case of the Gram-positive bacterium *S. aureus* ATCC 25923, the effect was bactericidal even with the 16-fold diluted original sample 3. In all other cases, no growth inhibition was observed, which can be explained by the masking effect of the protein-rich medium Meat-Peptone Broth (MPB), which chelates the nanoparticles and reduces their effect. The results are presented in Table 5.

## 4. Discussion

By TEM, SAED, and HRTEM analysis, the formation of gold nanoparticles and graphene structures in the obtained composites was confirmed (Figure 3, Figure 4, Figure 5 and Figure 6). The gold nanoparticles exhibited an individual size range between 2 and 10 nm; however, within the composite structure, they exhibit a tendency to form aggregates with a size range between 10 and 60 nm. These variations in size and distribution are significant because smaller nanoparticles generally have a higher surface area to volume ratio, which enhances catalytic activity and interaction with biological systems. The uniform distribution of these particles implies consistent performance across applications, such as in catalysis or drug delivery systems. The uniform size may contribute to predictable behavior in biological environments, potentially reducing variability in experimental outcomes.

HaCaT keratinocyte cells were used to study cell viability and morphology after treatment with different concentrations of RGO/AuNPs composites (Figure 7 and Figure 8). The investigation conducted highlighted that RGO/Au 0.500/0.175 g/L (sample 3) is the most cytotoxic nanomaterial among the five studied compositions. This high level of cytotoxicity could be advantageous for targeted cancer therapy, where controlled cell death is desired, especially when targeting tumor cells. However, such a cytotoxic effect may be limited in applications that require biocompatibility, such as wound healing or tissue engineering, where minimal toxicity is essential. Therefore, the specific application of this nanocomposite should be carefully considered to balance efficacy with safety.

Compared to literature, our composite achieved complete *E. coli* inhibition at only 0.175 g/L Au, whereas Turcheniuk et al. [7] used ≥0.5 g/L Au in hybrid structures for similar effects. Mehdipour-Ataei & Aram showed that RGO-based sensors with AuNPs required over 200 µg/mL to achieve partial antibacterial effects, especially against Gram-positive strains [8].

Our data also highlights the influence of the dispersion medium. When nanoparticles were suspended in saline, their bactericidal action was preserved even at high dilutions (Table 4), but in protein-rich MPB medium, the effect was significantly reduced (Table 5)—likely due to protein corona formation, which has been previously described to reduce nanoparticle–cell interactions [18]. Despite this, sample 3 retained a bactericidal effect even in MPB, completely inhibiting *S. aureus* at all dilutions. 

In our study, RGO and AuNPs did not show measurable antimicrobial activity when separate, confirming the synergistic effect when combined. This is visually evident in the inhibition zones shown in Figure 9, and aligns with observations by Turcheniuk et al., where individually applied RGO or AuNPs had limited impact, while combined systems displayed enhanced activity due to local plasmonic heating and membrane destabilization [7].

Liu et al. proposed similar mechanisms, showing that RGO induces membrane damage and oxidative stress, while metallic nanoparticles act as localized ROS generators [14]. Likewise, Tan et al. reported that RGO/Ag nanocomposites showed complete inhibition of *E. coli* at ~100 µg/mL, but partial inhibition of S. aureus, which is consistent with the Gram-positive cell wall acting as a physical barrier [13].

These results suggest a synergistic antibacterial mechanism, which can be attributed to mechanical membrane disruption by RGO nanosheets; ROS (reactive oxygen species) generation facilitated by AuNPs; electron transfer interactions between the graphene and metal nanoparticles; and increased local concentration of nanoparticles at bacterial surfaces due to aggregation.

The antibacterial efficacy of the synthesized RGO/AuNPs composites was evaluated using three bacterial strains (*E. coli*, *S. aureus* and *B. subtilis*), and the results showed distinct and promising activity profiles (Table 4 and Table 5; Figure 9 and Figure 10). The strongest bactericidal effect was observed for sample 3 (RGO/Au 0.500/0.175 g/L), which led to complete inhibition of *E. coli* even at 16× dilution and to a reduction in *S. aureus* CFU by over 8 logs (from 10^9^ to 10^1^ CFU/mL).

According to the agar diffusion methods, the best antibacterial activity against *B. subtilis* was observed from samples 2(RGO/Au 0.250/0.175 g/L) and 3 (RGO/Au 0.500/0.175 g/L), as shown in Figure 9. The enhanced antibacterial efficacy of this composition can be attributed to the synergistic action between reduced graphene oxide (RGO) and gold nanoparticles (AuNPs). The RGO layers likely contribute by causing mechanical disruption of bacterial membranes, while the AuNPs generate reactive oxygen species (ROS), which induce oxidative stress within bacterial cells. In addition to these mechanisms, recent studies suggest that electrochemical interactions between graphene layers and AuNPs may further enhance antibacterial activity. These interactions likely lead to increased ROS generation and altered membrane potential, which contributes to destabilizing the bacterial cell wall and impairing intracellular processes. The electrocatalytic properties of the RGO/AuNPs composite may also play a significant role, enhancing the redox reactions at the bacterial membrane and intensifying the bactericidal effect. This combined set of mechanisms significantly boosts the antibacterial potency of sample 2, effectively compromising the cell wall integrity and intracellular functions of *B. subtilis*, ultimately resulting in a substantial reduction in bacterial viability. Broth dilution tests show bactericidal activity of samples 2 and 3 in the case of saline dilution against *E. coli* and *S. aureus* (Table 4). Meat-peptone broth dilutions show activity in sample 3 (Table 5) which may be due to the chelation of the agents studied in the presence of peptides.

## 5. Conclusions

This study demonstrates the successful one-step synthesis of RGO/AuNP nanocomposites via a modified citrate method, enabling the simultaneous reduction of Au^3+^ and GO using sodium citrate as a green reducing agent. The resulting composites displayed well-dispersed AuNPs (10–60 nm) on RGO sheets, confirmed by TEM and SAED analysis. The obtained composites demonstrated synergistic antibacterial and dose-dependent cytotoxic activity at a record-low gold content, setting it apart from previously published studies in the field. Crystal violet assay with human keratinocyte cell lines has highlighted the performance of different ratios of RGO:AuNPs from most cytotoxic (RGO/Au 0.500/0.175 g/L) to most biocompatible (RGO/Au 0.125/0.175 g/L). Thus, fine-tuning for specific applications is proven possible by merely varying the composition. Agar tests show limited diffusion ability of the composites, which, when combined with the zeta potential studies, can be explained by the formation of larger, less mobile aggregates.

Compared to previously published systems, our composites:

- use lower metal content and are synthesized through a simpler, scalable, and greener one-step method;
- exhibit equal or superior biological performance, and are suitable for multifunctional biomedical applications, including antibacterial coatings and targeted therapies.

In terms of cytotoxicity, RGO/Au 0.500/0.175 g/L exhibited 57% reduction in HaCaT cell viability at high concentration (80 µL/100 µL), indicating potential for selective anti-tumor applications. Lower concentrations (1–25 µL) retained over 80% viability, suggesting acceptable biocompatibility in controlled doses. These values are in line with results by Tan et al., who reported similar IC_50_ thresholds in RGO/Ag systems [13].

Among the tested formulations, RGO/Au 0.500/0.175 g/L and RGO/Au 0.250/0.175 g/L exhibited the strongest antibacterial properties, leading to complete inhibition of *E. coli* and up to 8-log reduction in *S. aureus* at 16-fold dilutions. These results surpass the activity of RGO and AuNPs when examined separately, confirming the synergistic mechanism of action. Notably, full *E. coli* inhibition was achieved at only 0.175 g/L AuNPs, while comparable studies required ≥0.5 g/L AuNPs [7].

## Figures and Tables

**Table 1:** Composition of the obtained samples.

No.	Composition	RGO [g/L]	AuNPs [g/L]
Sample 1	RGO/Au 0.125/0.175 g/L	0.125	0.175
Sample 2	RGO/Au 0.250/0.175 g/L	0.250	0.175
Sample 3	RGO/Au 0.500/0.175 g/L	0.5	0.175
Sample 4	RGO 0.500 g/L	0.5	-
Sample 5	Au 0.175 g/L	-	0.175

**Table 2:** Miller indices and interplanar spacing of SAED detected phases.

COD	Spac. Gr.	Phase	hkl	D [Å]
#96-101-1061	P63mc	C 2H	100	2.1391
		C 2H	110	1.2350
#96-900-8464	Fm-3m	Au	111	2.3546
		Au	200	2.0391
		Au	222	1.1773

**Table 3:** Apparent size, polydispersity index and zeta potential of the tested samples.

PDI	Zeta Potential [mV]	Size [nm]	Composition
0.850 ± 0.007	–35.03 ± 0.67	248.5 ± 18.3	RGO/Au 0.125/0.175 g/L
0.240 ± 0.099	–36.96± 0.59	589.7 ± 25.7	RGO/Au 0.250/0.175 g/L
0.300 ± 0.117	–38.50 ± 1.18	564.8 ± 54.1	RGO/Au 0.500/0.175 g/L
0.433 ± 0.036	–36.84 ± 1.16	835.6 ± 45.9	RGO 0.500 g/L
0.402 ± 0.147	–27.72 ± 0.22	277.5 ± 7.5	Au 0.175 g/L

**Table 4:** Antibacterial properties of RGO/AuNPs against *S. aureus* and *E. coli* in saline.

16×	8×	4×	2×	Composition
			*Staphylococcus aureus*	
-	-	8.1 × 10^6^	1.72 × 10^7^	RGO/Au 0.125/0.175 g/L
1 × 10^9^	1 × 10^9^	1 × 10^9^	3 × 10	RGO/Au 0.250/0.175 g/L
4 × 10^1^	3 × 10^1^	2 × 10^1^	1 × 10	RGO/Au 0.500/0.175 g/L
1 × 10^9^	1 × 10^9^	1 × 10^9^	1 × 10^9^	RGO 0.500 g/L
-	-	1.39 × 10^7^	1.31 × 10^7^	Au 0.175 g/L
			*Escherichia coli*	
-	-	1.22 × 10^7^	1.76 × 10^7^	RGO/Au 0.125/0.175 g/L
0	0	0	0	RGO/Au 0.250/0.175 g/L
3 × 10^1^	0	0	0	RGO/Au 0.500/0.175 g/L
1 × 10^9^	1 × 10^9^	1 × 10^9^	1 × 10^9^	RGO 0.500 g/L
-	-	1.48 × 10^7^	1.67 × 10^7^	Au 0.175 g/L

**Table 5:** Antibacterial properties of RGO/AuNPs *S. aureus* and *E. coli* in MPB.

16×	8×	4×	2×	Composition
			*Staphylococcus aureus*	
1 × 10^9^	1 × 10^9^	1 × 10^9^	1 × 10^9^	RGO/Au 0.125/0.175 g/L
1 × 10^9^	1 × 10^9^	1 × 10^9^	1 × 10^9^	RGO/Au 0.250/0.175 g/L
0	0	0	0	RGO/Au 0.500/0.175 g/L
1 × 10^9^	1 × 10^9^	1 × 10^9^	1 × 10^9^	RGO 0.500 g/L
1 × 10^9^	1 × 10^9^	1 × 10^9^	1 × 10^9^	Au 0.175 g/L
			*Escherichia coli*	
1 × 10^9^	1 × 10^9^	1 × 10^9^	1 × 10^9^	RGO/Au 0.125/0.175 g/L
1 × 10^9^	1 × 10^9^	1 × 10^9^	1 × 10^9^	RGO/Au 0.250/0.175 g/L
5.7 × 10^6^	0	0	0	RGO/Au 0.500/0.175 g/L
1 × 10^9^	1 × 10^9^	1 × 10^9^	1 × 10^9^	RGO 0.500 g/L
1 × 10^9^	1 × 10^9^	1 × 10^9^	1 × 10^9^	Au 0.175 g/L

## Data Availability

The original contributions presented in this study are included in the article. Further inquiries can be directed to the corresponding author.

## Abbreviations

The following abbreviations are used in this manuscript:

GO	Graphene Oxide
RGO	Reduced Graphene Oxide
AuNPs	Gold nanoparticles
MPB	Meat-peptone broth
McF	McFarland standard
DLS	Dynamic Light Scattering
PDI	Polydispersity index
TEM	Transmission Electron Microscopy
HRTEM	High Resolution Transmission Electron Microscopy
SAED	Selected Area Electron Diffraction
HaCaT	Human keratinocyte cell culture
NBIMCC	Bulgarian national collection for microorganisms and cell cultures
FBS	Fetal Bovine Serum
